# M2a Macrophage-Secreted CHI3L1 Promotes Extracellular Matrix Metabolic Imbalances *via* Activation of IL-13Rα2/MAPK Pathway in Rat Intervertebral Disc Degeneration

**DOI:** 10.3389/fimmu.2021.666361

**Published:** 2021-06-08

**Authors:** Long Li, Kang Wei, Yifan Ding, Paerxiati Ahati, Haoran Xu, Huang Fang, Huan Wang

**Affiliations:** Department of Orthopedics, Tongji Hospital, Tongji Medical College, Huazhong University of Science and Technology, Wuhan, China

**Keywords:** intervertebral disc degeneration, M2a macrophage, nucleus pulposus cell, CHI3L1, MAPK

## Abstract

The accumulation of macrophages in degenerated discs is a common phenomenon. However, the roles and mechanisms of M2a macrophages in intervertebral disc degeneration (IDD) have not been illuminated. This study investigated the expression of the M2a macrophage marker (CD206) in human and rat intervertebral disc tissues by immunohistochemistry. To explore the roles of M2a macrophages in IDD, nucleus pulposus (NP) cells were co-cultured with M2a macrophages *in vitro*. To clarify whether the CHI3L1 protein mediates the effect of M2a macrophages on NP cells, siRNA was used to knock down CHI3L1 transcription. To elucidate the underlying mechanisms, NP cells were incubated with recombinant CHI3L1 proteins, then subjected to western blotting analysis of the IL-13Rα2 receptor and MAPK pathway. CD206-positive cells were detected in degenerated human and rat intervertebral disc tissues. Notably, M2a macrophages promoted the expression of catabolism genes (MMP-3 and MMP-9) and suppressed the expression of anabolism genes (aggrecan and collagen II) in NP cells. These effects were abrogated by CHI3L1 knockdown in M2a macrophages. Exposure to recombinant CHI3L1 promoted an extracellular matrix metabolic imbalance in NP cells *via* the IL-13Rα2 receptor, along with activation of the ERK and JNK MAPK signaling pathways. This study elucidated the roles of M2a macrophages in IDD and identified potential mechanisms for these effects.

## Introduction

Low back pain has been an quite common disorder (1), experienced by more than 500 million people worldwide. Lower back pain is the first leading cause of disability among individuals of working age. Intervertebral disc degeneration (IDD) (2), an age-related chronic pathological process, is generally regarded as a primary cause of this disease (3, 4).

An imbalance between the synthesis and degradation of extracellular matrix (ECM) secreted by NP cells has been identified as the main pathophysiological process of IDD (5). In addition, multiple studies have demonstrated the accumulation of macrophages in degenerated intervertebral disc tissues (6, 7). However, the potential roles of macrophages in the onset of IDD have not been fully elucidated (8, 9), partly because of substantial heterogeneity among macrophages. Understanding these roles is critical for developing new IDD treatments based on the underlying pathophysiology (10).

Macrophages exhibit multiple phenotypes (11), commonly divided into M1, M2a, and M2c subsets (12), identified by cell surface markers CCR7, CD206, and CD163 (13), respectively. Recently, Nakazawa et al. reported that all three types of macrophage markers were present in degenerated intervertebral disc tissues (14). Although several studies have tried to investigate the roles of macrophages in IDD (9), there has been minimal consideration of specific types of macrophage, especially M2a (CD206) macrophages (8).

In M2a macrophages, the expression of chitinase 3-like 1 protein (CHI3L1) is elevated, compared with other macrophage types (15). CHI3L1 is a secreted glycoprotein that can facilitate tumor invasion and metastasis by upregulating the expression levels of matrix metalloproteinase (MMP) genes in various tumor cells (16–18). In many inflammatory diseases (e.g., rheumatoid arthritis and liver cirrhosis), CHI3L1 is strongly expressed by macrophages and can mediate proinflammatory effects (19, 20). Thus, we hypothesized that M2a macrophages secrete CHI3L1, thus contributing to the pathological progression of IDD.

To test our hypothesis, we investigated the infiltration of M2a macrophages in human and rat degenerated NP tissues by means of immunohistochemistry. Subsequently, we induced M2a polarization from human monocyte THP-1 cells by stimulation with interleukin (IL)-4, which is the strongest inducer of the M2a phenotype (21). By using a co-culture system, we found that M2a macrophages exacerbated a metabolic imbalance in the ECM, partly through secretion of CHI3L1 protein. Finally, we found that IL-13Rα2 and the mitogen-activated protein kinase (MAPK) signaling pathway were involved in this process.

## Materials and Methods

### Clinical Sample Collection and Ethical Considerations

The study was approved by the Ethics Committee of Tongji Hospital, Tongji Medical College, Huazhong University of Science and Technology. All patients signed informed consent forms. In total, 18 human intervertebral disc NP tissues were collected from surgeries in which intervertebral disc resection was required. Among the samples, 10 were obtained from patients with IDD (IDD group, seven female patients and three male patients; mean age = 52 ± 8 years) and eight were obtained from patients with adolescent idiopathic scoliosis or congenital scoliosis without signs of disc degeneration (22) (normal group, five female patients and three male patients; mean age = 15 ± 6 years).

### Animal Model

The animal study was reviewed and approved by Ethics Committee of Tongji Hospital, Tongji Medical College, Huazhong University of Science and Technology. Sprague–Dawley rats were purchased from the Hubei Province Experimental Animal Center (Wuhan, China). A rat IDD model was established in the coccygeal vertebra through needle puncture, as described previously (23). Briefly, 12 Sprague–Dawley rats (6–8 weeks old, female) were randomly divided into normal and IDD model groups (n = 6 rats per group). After induction of anesthesia, discs corresponding to Co 8/9 caudal vertebral gaps were identified as puncture points. A 20-G puncture needle was inserted into the rat tail until complete penetration was achieved, rotated 360° and maintained for 30 s, and then removed. Two months after surgery, magnetic resonance imaging was performed to confirm that degeneration modeling had been successful.

### Sample Processing and Immunohistochemistry

Rat NP tissues and a small portion of adjacent vertebrae were harvested 2 months after surgery. After tissues had been fixed for 24 h in 10% formaldehyde, they were soaked in ethylenediaminetetraacetic acid decalcifying solution for 2 months and embedded in paraffin. Human NP tissues were fixed in 4% paraformaldehyde for 2 h before paraffin embedding. All paraffin-embedded tissues were cutted into 5-µm sections for subsequent immunohistochemical analysis.

Immunohistochemical staining was performed to examine CD206 expression in human or rat NP tissues, as described previously (24). Briefly, slices were deparaffinized for 15 min in xylene and dehydrated in a graded ethanol series (from 99% to 75%) for 20 min per solution. For antigen retrieval, sections were incubated in 0.01 M citrate buffer (pH 6.0) for 20 min at 95°C. Endogenous peroxidases were inhibited by incubation in 3% H2O2 at 37°C for 10 min. After blocking with goat serum for 30 min at room temperature, primary anti-CD206 antibody (ab64693, Abcam, Cambridge, UK) was incubated overnight at 4°C. After incubation with horseradish peroxidase-conjugated goat anti-rabbit IgG for 1 h at room temperature, cellular nuclei staining was performed by diaminobenzidine treatment and counterstaining with hematoxylin for 3 min. In between all incubation steps, sections were washed three times with phosphate-buffered saline (PBS; 5 min per wash). Finally, slices were photographed under a light microscope. The ratio of positive cells and the mean optical density were analyzed with ImageJ software, version 2.0.

### Rat NP Cells Extraction and Treatment

Rat NP cells were isolated from the coccygeal intervertebral discs of Sprague–Dawley rats (6–8 week old) as described before (25). Briefly, gelatinous NP tissues were isolated under aseptic conditions and then digested in 0.25% trypsin for 30 min and 0.2% type II collagenase for 3 h at 37°C. After sufficient digestion, the tissues were suspended in DMEM/F12 medium (HyClone, Logan, UT, USA) containing 10% fetal bovine serum (HyClone) and 1% streptomycin/penicillin, then incubated at 37°C in a humidified 5% CO_2_ incubator. Passages were conducted after cell fusion and the first three passages of cells were used for subsequent experiments.

### M2a Macrophage Polarization and Co-Culture

Human THP-1 monocytes were differentiated into M0 or M2a macrophages, as described previously (26, 27). Briefly, human monocyte cell line THP-1 cells were stimulated with 100 ng/ml phorbol 12-myristate 13-acetate (PMA, P1585, Sigma, USA) for 12 h to achieve M0 polarization. Subsequently, M0 macrophages were incubated continually with 20 ng/ml of human recombinant IL-4 (200-04, PeproTech, USA) for 24 h to generate M2a macrophages. Using a Transwell co-culture system (0.4-μm pore size, Costar, Corning, NY, USA), M2a macrophages and NP cells were co-cultured for 48 h.

### Immunofluorescence

After fixing with 4% paraformaldehyde for 15 minutes, treated cells were permeabilized and blocked with 5% bovine serum albumin/0.3% Triton™ X-100 PBS for 1 h at room temperature. Primary antibodies against CD206 (ab64693, Abcam), CHI3L1 (ab180569, Abcam), or IL-13Rα2 (ab108534, Abcam) were applied and incubated overnight at 4°C. Secondary antibody were incubated with Cy3‐conjugated goat anti‐rabbit IgG (1:100, BA1032, Boster, Wuhan, China) or fluorescein isothiocyanate‐conjugated goat anti-mouse IgG (1:100, BA1101, Boster) for 1 h. For nuclear counterstaining, DAPI was incubated for 10 min. Cells were protected from light during incubation with secondary antibodies and DAPI. In between all incubation steps, cells were washed three times with PBS (5 min per wash). Cells were examined and photographed by fluorescence microscopy.

### siRNA Knockdown Experiments

Knockdown of the CHI3L1 gene in M2a macrophages or the IL-13Rα2 gene in NP cells was achieved by transfection with siRNA (targeting CHI3L1 or IL-13Rα2, or a scrambled sequence as negative control), using Lipofectamine 2000 (Invitrogen, USA) (28). siRNA sequences were as follows: CHI3L1, 5’-CCAUAUCAUCUACAGCUUUTT-3’ and 5’-AAAGCUGUAGAUGAUAUGGGT-3’; IL-13Rα2, 5’-GGGUUAUCUCUAUUUGCAATT-3’ and 5’ UUGCAAAUAGAGAUAACCCAG-3’. Knockdown efficacy was detected by western blotting.

### Western Blotting Analysis

Cellular proteins (25 μg) were separated and probed with primary antibodies to MMP3 (17873-1-AP, Proteintech), MMP9 (10375-2-AP, Proteintech), COL2A1 (15943-1-AP, Proteintech), aggrecan (13880-1-AP, Proteintech), phospho‐JNK (#4668, CST), JNK (#9252, CST), phospho‐ERK (#4370, CST), ERK (#4695, CST), phospho‐p38 (#4511, CST), p38 (#8690, CST), and GAPDH (60004-1-Ig, Proteintech). Following incubation with primary antibody overnight at 4°C, HRP-conjugated anti-rabbit or anti-mouse secondary antibodies (BA1054, BA1050, Boster) were incubated; protein bands were visualized using an enhanced chemiluminescence system (Thermo Fisher Scientific) (29). Quantification of western blotting results was performed using ImageJ software, version 2.0.

### Quantitative Reverse-Transcription PCR (qRT-PCR)

Total RNA was isolated with TRIzol reagent (Invitrogen) and cDNA was reverse transcribed using the Reverse Transcription Kit (Toyobo, Japan). The qRT-PCR experiments were performed on a Bio-Rad iQ5 real-time PCR detection system using SYBR Green reagent (Toyobo) (30). Primer sequences are listed in **Table 1**.

**Table 1:** Primers used in this study.

Gene	Species	Forward	Reverse
CD206	Human	aacggactgggttgctatca	cccgatcccttgtagagcat
CD301	Human	gctgctggtcatcatctgtg	cctccacctcagctttcaga
Arginase1	Human	gtggaagaaggccctacagt	gcttttcccacagaccttgg
GAPDH	Human	ccaaggagtaagacccctgg	tggttgagcacagggtactt
MMP3	Rat	atgacagggaagctggactc	ctggagaatgtgagtggggt
MMP9	Rat	aggatggtctactggcacac	gtgcaggacaaataggagcg
COL2A1	Rat	tgttgacattgcacccatgg	cagccattcagtgcagatcc
Aggrecan	Rat	catgcatcctgtgaccactg	gcatcacttcacagcggtag
GAPDH	Rat	agacagccgcatcttcttgt	cttgccgtgggtagagtcat

### Statistical Analysis

GraphPad Prism 5 software (GraphPad Inc., La Jolla, CA, USA) was used for statistical analysis. All quantitative results are shown as means ± standard deviations. Data between two groups were compared using the Student’s t -test. One-way analysis of variance (ANOVA) was used to compare data among three or more groups. Percentages were compared by Fisher’s exact test using R software, version 3.5.3. A p value < 0.01 was regarded as statistically significant.

## Results

### Expression of the M2a Macrophage Marker, CD206, in Human and Rat Degenerated Intervertebral Disc Tissues

**Figure 1:**
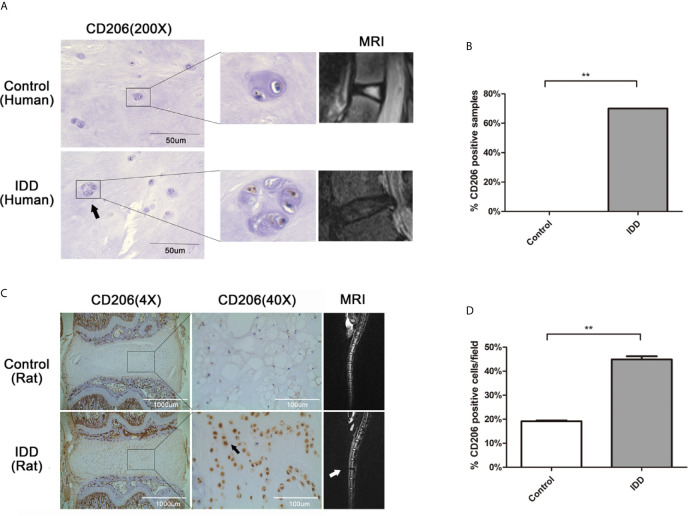
Expression of the M2a macrophage marker, CD206, in human and rat degenerated intervertebral disc tissues. **(A)** Immunolocalization of CD206-positive cells in human intervertebral disc tissues. CD206-positive cells were detected using immunofluorescence staining with an anti-CD206 antibody. Representative pictures are from a patient with congenital scoliosis (11-year-old girl) and a patient with IDD (56-year-old woman), representing normal and IDD samples, respectively. **(B)** The ratio of CD206-positive samples between normal and IDD samples. CD206-positive cells were absent from normal disc samples (0/8, 0%), although seven of the 10 IDD samples exhibited at least one CD206-positive cell (7/10, 70%); Fisher’s exact test, **p < 0.01. **(C)** Immunolocalization of CD206-positive cells in rat intervertebral disc tissues. Intervertebral disc degeneration in a rat model was induced by needle puncture, and immunohistochemical staining of intervertebral disc tissues was performed with an anti-CD206 antibody. **(D)** Quantitative analysis of CD206 expression, the dates were analyzed by Student’s t -test. Significant differences between groups are shown as **p < 0.01.

To evaluate the accumulation of M2a macrophages in degenerative intervertebral disc tissues, immunohistochemical analysis of CD206 (a surface marker for M2a) was performed using human and rat intervertebral disc tissues. Analysis of human intervertebral disc tissues showed that CD206-positive cells were absent from normal disc samples (0/8, 0%), although seven of the 10 IDD samples exhibited at least one CD206-positive cell (7/10, 70%) (**Figures 1A, B**). Analysis of rat intervertebral disc tissues showed significantly elevated expression of CD206 in degenerated intervertebral disc tissues, compared with the normal group (**Figures 1C, D**). Furthermore, CD206-positive cells in rat discs closely resembled resident NP cells, indicating that NP cells may also express marker of M2a macrophages. These findings indicated that significantly greater numbers of CD206-positive cells were present in degenerated disc tissues, comparing with normal group.

### Contribution of M2a Macrophages to ECM Metabolic Imbalance in NP Cells

**Figure 2:**
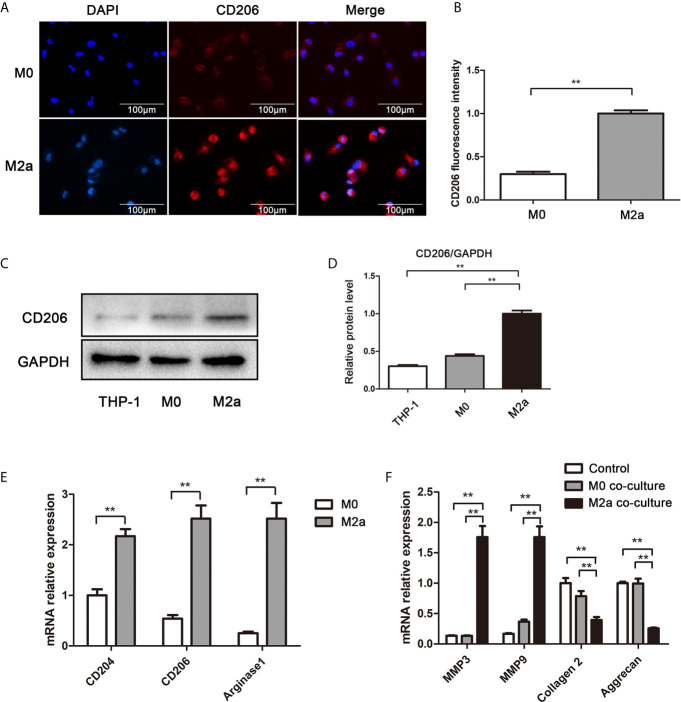
M2a macrophages promoted ECM degradation in NP cells. **(A)** Immunofluorescence analysis of the CD206 differentiation marker in M2a macrophages. M0 and M2a macrophages were differentiated from THP-1 monocytes by stimulation with phorbol 12-myristate 13-acetate or IL-4, respectively. Immunofluorescence staining for CD206 was performed. Scalebar, 100 µm. **(B)** Semi-quantitative analysis of CD206 immunofluorescence staining results. **(C)** Western blotting analysis of CD206 differentiation marker in M2a macrophages. M0 and M2a macrophages were differentiated from THP-1 monocytes. The protein levels of CD206 in THP-1, M0 (i.e., control), and M2a macrophages were determined by western blotting. **(D)** Quantification of CD206 protein levels in western blots. **(E)** qRT-PCR analysis of M2a macrophage differentiation marker genes (CD204, CD206, and arginase-1). **(F)** Effects of M2a macrophages on NP cells *via* Transwell co-culture. M0 and M2a macrophages were differentiated from THP-1 monocytes, then co-cultured with NP cells for 48 h. MMP3, MMP9, collagen II, and aggrecan expression levels of NP cells were analyzed by qRT-PCR. Experiments were repeated three times in triplicate. The dates were analyzed by One-way analysis of variance (ANOVA). Statistically significant difference: **P < 0.01.

To explore the effects of M2a macrophages on ECM metabolism, the macrophages were co-cultured with NP cells. Firstly, THP-1 monocytes were polarized into M0 (i.e., control) and M2a macrophages. Immunofluorescence staining revealed that the expression levels of CD206 were significantly elevated in M2a macrophages, compared with the levels in M0 macrophages (**Figures 2A, B**). Western blotting results confirmed this difference in expression levels (**Figures 2C, D**). To confirm M2a macrophage polarization, the mRNA expression levels of M2a-associated genes were measured by qRT-PCR. Importantly, the expression levels of CD204, CD206, and arginase-1 were significantly upregulated in M2a macrophages (**Figure 2E**). After successful polarization had been confirmed, M0 and M2a macrophages were co-cultured with NP cells. After 48 h of co-culture, the expression levels of anabolism genes (aggrecan and collagen II) and catabolism genes (MMP-3 and MMP-9) were detected by qRT-PCR. The results showed enhanced transcription of anabolism genes and reduced expression of catabolism genes in the M2a co-culture group, compared with the untreated and M0 co-culture groups (**Figure 2F**). Thus, M2a macrophages promoted ECM metabolic imbalance in NP cells.

### Role of CHI3L1 in Induction of ECM Metabolic Imbalance by M2a Macrophages

**Figure 3:**
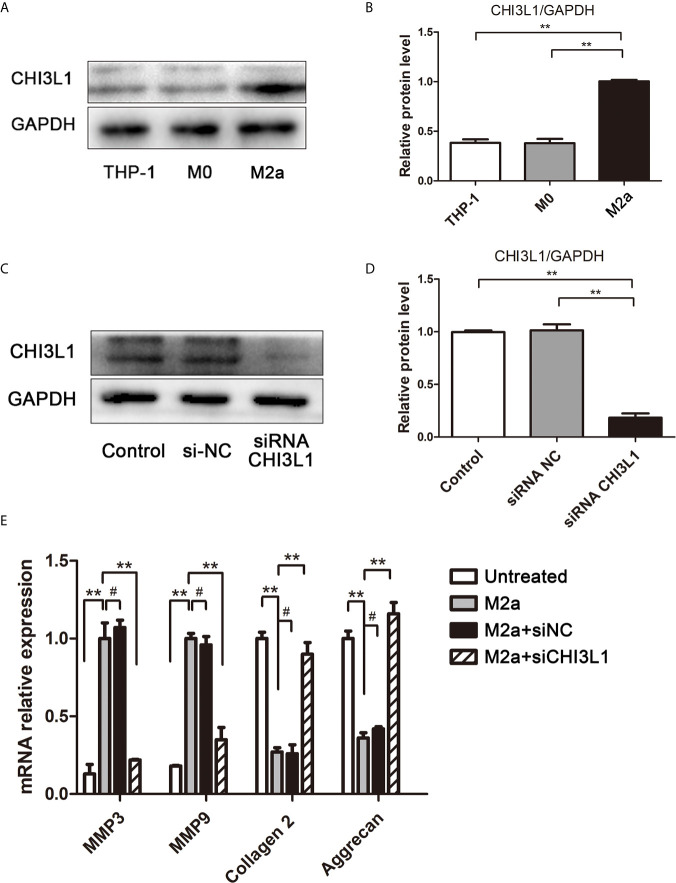
CHI3L1 mediated M2a macrophage-induced ECM degradation. **(A)** Expression of CHI3L1 in M2a macrophages. M0 and M2a macrophages were differentiated from THP-1 monocytes. The protein levels of CHI3L1 in THP-1, M0 and M2a macrophages were detected by western blotting. **(B)** Quantification of CHI3L1 protein levels in western blots. **(C)** siRNA silencing of CHI3L1 in M2a macrophages. After transfection with NC siRNA or CHI3L1 siRNA, the knockdown of CHI3L1 in M2a macrophages was confirmed by western blotting. **(D)** Quantification of CHI3L1 protein levels in western blots. **(E)** Effects of CHI3L1 silencing in M2a macrophages on ECM degradation in NP cells. After co-culture with CHI3L1-silenced M2a macrophages, the expression levels of MMP3, MMP9, collagen II and aggrecan in NP cells were analyzed by qRT-PCR. Experiments were repeated three times in triplicate. The dates were analyzed by One-way analysis of variance (ANOVA). Significant differences between groups are shown as **p < 0.01. #, no statistical difference.

To determine whether CHI3L1 mediated M2a macrophage-induced ECM metabolic imbalance in NP cells, CHI3L1 secretion was investigated in M2a macrophages. Western blotting analysis showed high expression of the CHI3L1 protein in M2a macrophages (**Figures 3A, B**). Subsequently, CHI3L1 gene expression in M2a macrophages was silenced by CHI3L1-siRNA, and effective knockdown was confirmed by western blotting (**Figures 3C, D**). The results showed that CHI3L1 knockdown abolished the M2a macrophage-induced ECM metabolic imbalance (**Figure 3E**). Taken together, these findings implied that CHI3L1 mediated M2a macrophage-induced ECM metabolic imbalance.

### Effects of Recombinant CHI3L1 (rCHI3L1) on ECM Degradation

**Figure 4:**
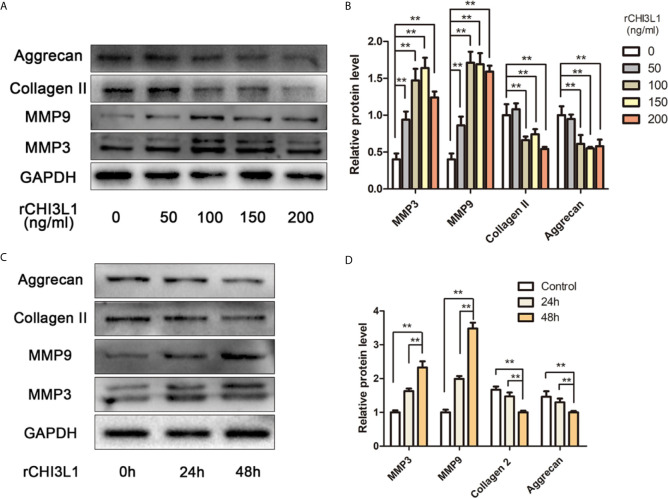
rCHI3L1 promoted ECM metabolism in NP cells. **(A)** Effects of rCHI3L1 concentration gradient on ECM metabolism in NP cells. Following incubation with different doses of rCHI3L1 for 48 h, the protein levels of MMP3, MMP9, collagen II and aggrecan in NP cells were detected by western blotting. **(B)** Quantification of the protein levels in **(A, C)** Effects of rCHI3L1 on ECM metabolism at different time points. NP cells were treated with rCHI3L1 (150 ng/ml) for 24 or 48 h. The protein levels of MMP3, MMP9, collagen II and aggrecan were detected by western blotting. **(D)** Quantification of the protein levels in **(C)** Data are presented as means ± standard deviations (n = 3) from one representative experiment of three independent experiments performed in triplicate. The dates were analyzed by One-way analysis of variance (ANOVA). Significant differences between groups are shown as **p < 0.01.

To explore further the effects of CHI3L1 on ECM metabolism, rCHI3L1 was added to NP cells at different concentrations or for different intervals. Notably, rCHI3L1 significantly upregulated the expression of catabolic genes MMP3 and MMP9, and downregulated the expression of anabolism genes aggrecan and collagen II (**Figure 4**). These effects were concentration- and time-dependent.

### Role of IL-13Rα2 in CHI3L1-Induced ECM Degradation in NP Cells

**Figure 5:**
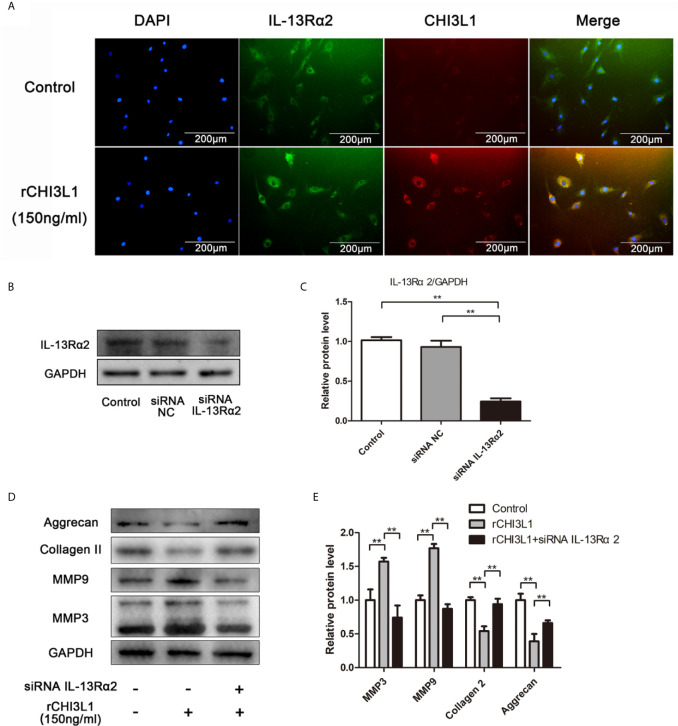
IL-13Rα2 was involved in CHI3L1-induced ECM metabolism. **(A)** Co-localization of CHI3L1 and IL-13Rα2 proteins in NP cells. After treatment with rCHI3L1 (150 ng/ml) for 24 h double immunofluorescence staining was performed on NP cells with anti-CHI3L1 and anti-IL-13Rα2 antibodies. **(B)** IL-13Rα2 was silenced in NP cells by siRNA. NP cells were transfected with NC siRNA or IL-13Rα2 siRNA, then grown for 24 h. Knockdown of IL-13Rα2 was confirmed by western blotting. **(C)** Quantification of the protein levels of IL-13Rα2 in western blots. **(D)** Effects of IL-13Rα2 silencing on CHI3L1-induced ECM metabolism. After transfecting with NC siRNA or IL-13Rα2 siRNA for 24 h, NP cells were incubated with rCHI3L1 (150 ng/ml) for another 24 h. The protein levels of MMP3, MMP9, collagen II, and aggrecan were detected by western blotting. **(E)** Quantification of the protein levels in **(D)**. Data are presented as means ± standard deviations (n = 3) from one representative experiment of three independent experiments performed in triplicate. The dates were analyzed by One-way analysis of variance (ANOVA). Significant differences between groups are shown as **p < 0.01.

To clarify the mechanism responsible for the effects of CHI3L1 on NP cells, a potential receptor for CHI3L1, IL-13Rα2, was then investigated. After treatment with rCHI3L1, double immunofluorescence staining showed co-localization of CHI3L1 and IL-13Rα2 in NP cells (**Figure 5A**). To explore further whether the IL-13Rα2 receptor was involved in ECM metabolism, IL-13Rα2 gene expression in NP cells was silenced by IL-13Rα2-siRNA, and successful IL-13Rα2 knockdown was confirmed by western blotting (**Figures 5B, C**). IL-13Rα2-knockdown NP cells were then incubated with rCHI3L1 and western blotting analysis showed that IL-13Rα2 receptor knockdown blocked the rCHI3L1-induced ECM metabolic imbalance in NP cells (**Figures 5D, E**). These results suggest that IL-13Rα2 may function as a receptor and is involved in CHI3L1-induced ECM degradation in NP cells.

### Mechanism Underlying Contribution of CHI3L1 to ECM Degradation

**Figure 6:**
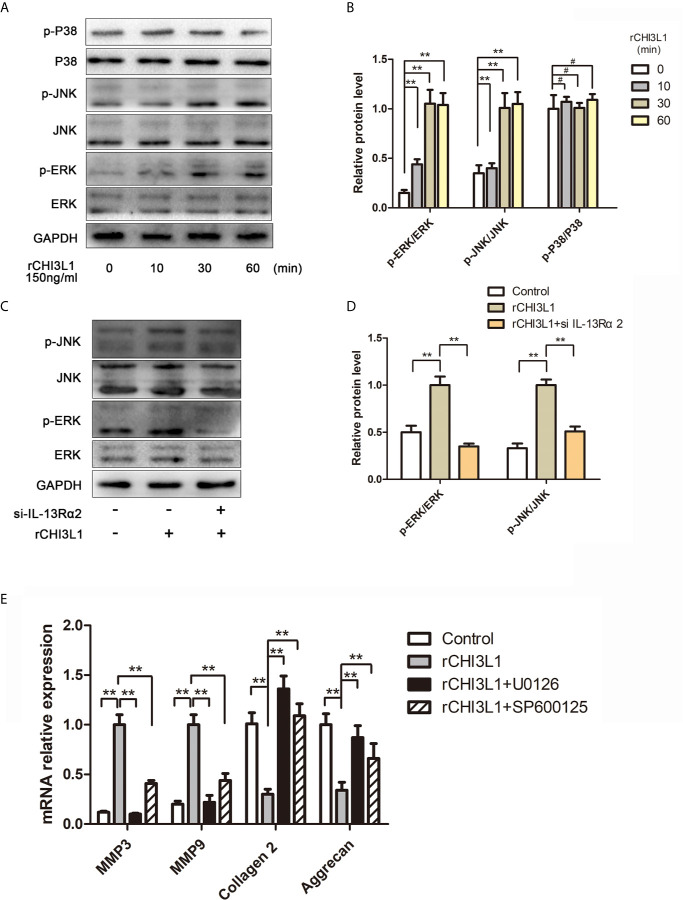
CHI3L1 promoted ECM degradation through the MAPK pathway in NP cells. **(A)** CHI3L1 affected the MAPK pathway at different time points. NP cells were incubated with rCHI3L1 (150 ng/ml) at different time points (10, 30, and 60 min). The MAPK activation was detected by western blotting. **(B)** Quantification of the protein levels in **(A, C)** IL-13Rα2 was involved in the CHI3L1-induced activation of the MAPK pathway. The IL-13Rα2-silenced NP cells were incubated with rCHI3L1 (150 ng/ml) for 60 min. The MAPK pathway related proteins were detected by western blotting. **(D)** Quantification of the protein levels in **(C). (E)** The MAPK signaling pathway was involved in CHI3L1-induced ECM degradation. After treatment with U0126 (ERK inhibitor) or SP600125 (JNK inhibitor) at 10 µM for 6 h, NP cells were incubated with rCHI3L1 (150 ng/ml) for 24 h. MMP3, MMP9, collagen II, and aggrecan expression levels were analyzed by qRT-PCR. Data are presented as means ± standard deviations (n = 3) from one representative experiment of three independent experiments performed in triplicate. The dates were analyzed by One-way analysis of variance (ANOVA). Significant differences between groups are shown as **p < 0.01. #, no statistical difference.

To characterize further the mechanism underlying the effects of CHI3L1 on ECM metabolism in NP cells, we investigated MAPK pathway activation. Western blotting analysis indicated that rCHI3L1 markedly induced the phosphorylation of ERK and JNK, although there was no substantial change in p38 phosphorylation (**Figures 6A, B**). Furthermore, western blotting analysis showed that IL-13Rα2 receptor blockage suppressed MAPK signaling pathway activation (**Figures 6C, D**). In addition, exposure to the ERK inhibitor (U0126, 10 nM) or JNK inhibitor (SP600125, 10 nM) blocked rCHI3L1-induced ECM metabolic imbalance (**Figure 6E**). In summary, these results demonstrate that the effects of CHI3L1 on NP cells are specifically mediated by the ERK and JNK pathways, but not the p38 pathway.

## Discussion

**Figure 7:**
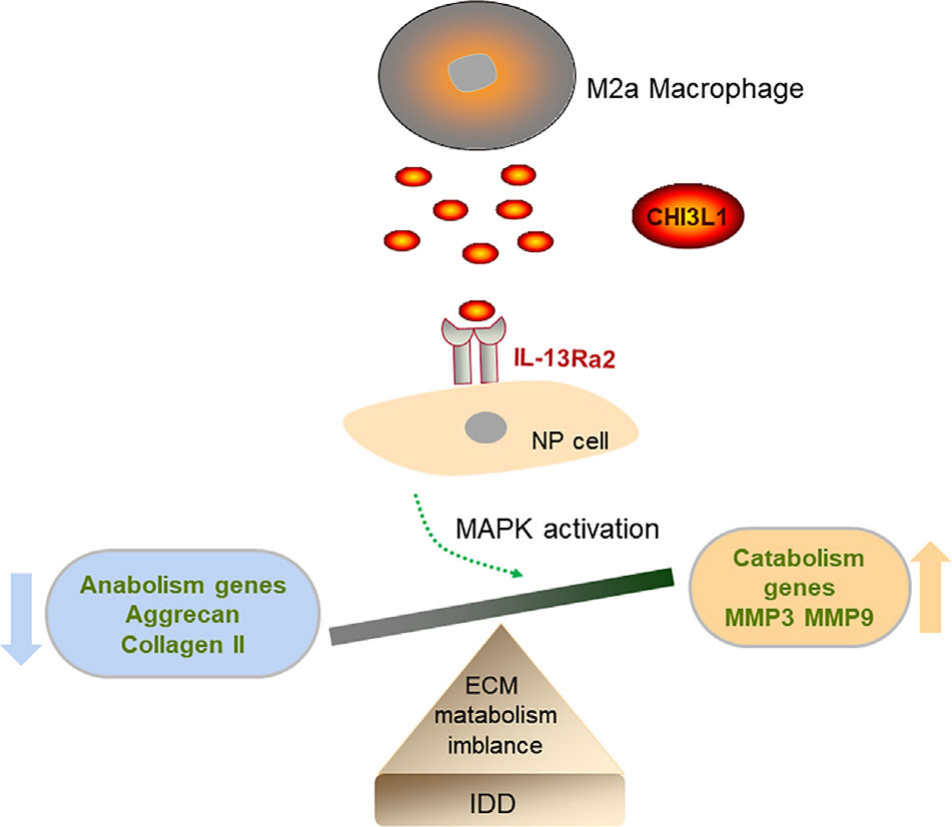
Diagram of the possible mechanism that M2a macrophage promotes intervertebral disc degeneration (IDD). M2a macrophage-secreted CHI3L1 promoted the expression of catabolism genes (MMP-3 and MMP-9) and suppressed the expression of anabolism genes (aggrecan and collagen II) in NP cells *via* activation of IL-13Rα2/MAPK pathway.

In this study, we confirmed the infiltration of M2a macrophages in human and rat degenerated NP tissues. Furthermore, Transwell co-culture experiments demonstrated that M2a macrophages promoted ECM metabolic imbalance in NP cells, at least partly, by secretion of CHI3L1 proteins. Our findings suggested that CHI3L1 acted *via* IL-13Rα2 receptors, as well as ERK and JNK signaling pathways. Our results provide insights concerning the effects of M2a macrophages on ECM metabolism in NP cells, as well as potential underlying mechanisms (**Figure 7**).

Although there has been extensive investigation of macrophage infiltration in disc tissues (31), there has been minimal research regarding specific types of macrophages in this process (32). To our knowledge, this is the first report to explore the effects of M2a macrophages in IDD. Also, we have not found substantial reports concerning the involvement of specific molecules or macrophage components (33). As a macrophage-secreted protein, CHI3L1 has been widely researched in various tumors (17, 18). Consistent with the findings in studies of tumors, we found that M2a macrophages secreted CHI3L1 proteins, thereby promoting ECM degradation. However, we noted that our findings were different from those of Wang et al. (34), who recently suggested that CHI3L1 may be a protective factor against IDD. In their experiments, CHI3L1 overexpression or knockdown was performed in NP cells to explore the role of CHI3L1 expression in those cells. In contrast, we mainly studied M2a macrophage-secreted CHI3L1 protein. This important difference in the source of CHI3L1 may explain the discrepancy between our findings and the results in previous reports.

IL-13Rα2 is an important receptor for CHI3L1 and has been extensively studied in the contexts of inflammation, tissue remodeling, and tumors (35, 36). This report is the first description of IL-13Rα2 involvement in IDD. Furthermore, explorations of downstream MAPK signaling pathways suggested that the ERK and JNK pathways, but not the p38 pathway, mediated these processes.

There were some limitations in this study. First, it was difficult to obtain completely normal disc tissues from surgery. This study used the most common approach, which involves acquisition of relatively normal disc tissues from patients with adolescent idiopathic scoliosis or congenital scoliosis without known or macroscopically evident disc pathology (22). Second, the macrophages and disc cells from different species, to some extent, may affect the results. On the other hand, CHI3L1 is highly conserved among species, which may partially mitigate the impact. Third, this study did not investigate the expression of macrophage markers by resident NP cells. Notably, Jones et al. found that endogenous disc cells could undergo differentiation to become phagocytes, including the development of phagocytic capacity (37). Here, we identified CD206-positive resident NP cells in the rat disc degeneration model. Therefore, it may be difficult to distinguish macrophage-like NP cells from true macrophages in intervertebral disc tissues, which requires additional investigation.

In conclusion, this is the first study to explore the roles of M2a macrophages in IDD and the underlying mechanisms of these effects. The results provide additional insights concerning IDD pathophysiology, which may aid in the development of targeted therapeutic strategies.

## Data Availability Statement

The original contributions presented in the study are included in the article/supplementary materials, further inquiries can be directed to: fanghuangtjh@126.com.

## Ethics Statement

The studies involving human participants were reviewed and approved by Ethics Committee of Tongji Hospital, Tongji Medical College, Huazhong University of Science and Technology. Written informed consent to participate in this study was provided by the participants’ legal guardian/next of kin. The animal study was reviewed and approved by Ethics Committee of Tongji Hospital, Tongji Medical College, Huazhong University of Science and Technology.

## Author Contributions

LL, KW, and HW conceived the project, designed the experiments and wrote the paper. LL, KW, and YD performed the experiments, analyzed the data. HF reviewed and edited the paper. HX and PA helped to perform the animal experiments and imaging experiments. All authors contributed to the article and approved the submitted version.

## Funding

This research was funded by the National Natural Science Foundation of China, grant number 81271347.

## Conflict of Interest

The authors declare that the research was conducted in the absence of any commercial or financial relationships that could be construed as a potential conflict of interest.

## Abbreviations

IDD, Intervertebral disc degeneration; NP, Nucleus pulposus; ECM, Extracellular matrix; MAPK, Mitogen-activated protein kinase.
